# Quantitative Pedagogy: A Digital Two Player Game to Examine Communicative Competence

**DOI:** 10.1371/journal.pone.0142579

**Published:** 2015-11-10

**Authors:** Matías Lopez-Rosenfeld, Facundo Carrillo, Gerry Garbulsky, Diego Fernandez Slezak, Mariano Sigman

**Affiliations:** 1 Laboratorio de Inteligencia Artificial Aplicada, Departamento de Computación, Facultad de Ciencias Exactas y Naturales, Universidad de Buenos Aires, Pabellón 1, Ciudad Universitaria, 1428 Buenos Aires, Argentina; 2 El Mundo de las Ideas, Buenos Aires, Argentina; 3 Laboratorio de Neurociencia, Universidad Torcuato Di Tella, Av. Figueroa Alcorta 7350, (C1428BCW) Ciudad de Buenos Aires, Argentina; University of Georgia, UNITED STATES

## Abstract

Inner concepts are much richer than the words that describe them. Our general objective is to inquire what are the best procedures to communicate conceptual knowledge. We construct a simplified and controlled setup emulating important variables of pedagogy amenable to quantitative analysis. To this aim, we designed a game inspired in Chinese Whispers, to investigate which attributes of a description affect its capacity to faithfully convey an image. This is a two player game: an emitter and a receiver. The emitter was shown a simple geometric figure and was asked to describe it in words. He was informed that this description would be passed to the receiver who had to replicate the drawing from this description. We capitalized on vast data obtained from an android app to quantify the effect of different aspects of a description on communication precision. We show that descriptions more effectively communicate an image when they are coherent and when they are procedural. Instead, the creativity, the use of metaphors and the use of mathematical concepts do not affect its fidelity.

## Introduction

*“Leaving hopes and utopias apart, probably the most lucid ever written about language are the following words by Chesterton: He knows that there are in the soul tints more bewildering, more numberless, and more nameless than the colours of an autumn forest… Yet he seriously believes that these things can every one of them, in all their tones and semitones, in all their blends and unions, be accurately represented by an arbitrary system of grunts and squeals. He believes that an ordinary civilized stockbroker can really produce out of this own inside noises which denote all the mysteries of memory and all the agonies of desire.”*

Jorge Luis Borges (The analytical language of John Wilkins)

Chinese whispers, also known as broken telephone, is a game in which a message is passed through a line of people. The last player announces the message which is often very different from the one that was uttered by the first. The game illustrates how different sources of noise accumulate, contaminating verbal communication.

However, even in the absence of noise, language poses a structural limitation in human communication. As expressed by JL Borges in the quote above, inner concepts are much richer than the words that describe them. Moreover, the same words often construct very distinct mental representations in different individuals. This is, of course, an essential problem of pedagogy: how to faithfully communicate concepts using words.

This abilitiy is often refered as *Communicative competence*, i.e. the grammatical knowledge and skill of use as well as the social adaptation in communication [1]. Chomsky pointed out two related concepts: competence and performance, that is the grammatical and social knowledge and the effectiveness of the communication process given the previous competence [2]. These terms—Competence and performance—have been widely studied in the learning and teaching of second language [3, 4].

One of the most popular ways to evaluate the communicative effectiveness is the language tests. The applied linguistics literature has deeply detailed how to design and quantify language tests [5, 6]. How to assess each area of language, e.g. reading, writing, or communication for specific purposes, has its own difficulties. Moreover, how to measure tests and interpret quantitative results remains as an open problem, e.g. Luoma’s book on speaking assessment [7].

**Fig 1:**
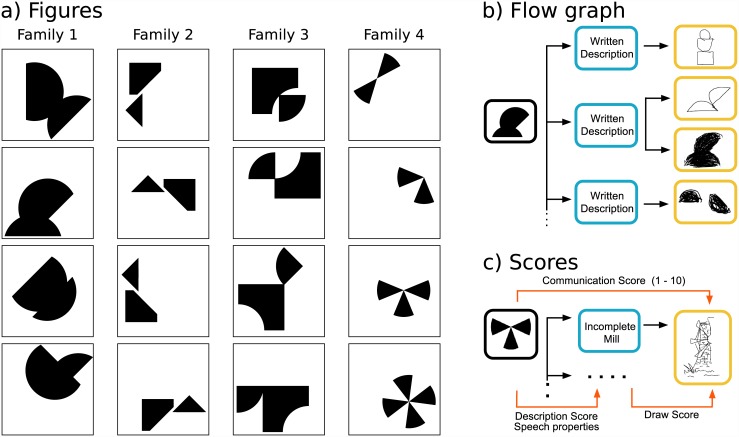
Flow graph of the game. The panel a) shows the 4 different families of images. Each familiy is composed of 4 variations of the same image. Panel b) shows a different possible flowgraphs. Starting with a real image example, from this image, many different subjects created different written descriptions, with one of these as instruction, other distinct subjects drew different pictures. Panel c) shows the three differents scores: 1) Communication Score: visual similarity among an image and a drawn picture. 2) Description Score: the quality of the written text as instruction to replay the image 3) Draw Score: the *power of the explanation* for the written description concerning the drawn picture. Beside these scores, the subjects rated pairs of image-written text some *Speech properties*.

Instead, we propose a completely different approach by quantifying the communicative competence and performance by crowdsourcing the evaluation process. Motivated by Games With A Purpose (GWAP)[8], we measure both competence and performance asking people to vote this abilities in a game. Here we designed a game inspired in Chinese Whispers, to investigate which attributes of a description affect its capacity to faithfully convey an image. This is a two player game: an emitter and a receiver. The game consisted in three phases (see Fig 1). First, the emitter was shown a simple geometric figure (see Fig 1a) and was asked to describe it in words. Second, she was informed that this description would be passed to the receiver who had to replicate the drawing form this description (see Fig 1b). Also, she received a description from another emitter having to replicate the original drawing.

Several studies have shown that images with high emotional content capture more attention than neutral ones (e.g. [9–11]). To avoid the interference with these *distractors*, we selected pictures with geometrical figures, which are pictures with neutral emotion (see Fig 1a).

Once the writing and drawing phases were completed, participants were asked to rank different stages of the communication process of other players (see Fig 1c): 1) the quality of the description of the geometric figure produced by the emitter (“*Description score*” or communication competence), 2) the quality of the drawing produced by the receiver given the description that he obtained (“*Drawing score*”), 3) the visual similarity of the original figure and the resulting drawing (“*Communication score*” or communication performance).

To assess the performance of a description, participants ranked five different speech properties of each description:

- Its creativity. There is a growing belief in the general public that creativity [12] is a fundamental aspect of effective human communication. Here we examine this in a quantitative manner to investigate whether –for this narrow class of human communication– the perceived creativity of a description correlates with the success to effectively communicate a message.
- Use of metaphors. Metaphors is vastly used in the educational discourse, particularly in teaching the common sense in scientific knowledge [13]. We investigate how this description strategy performs in communicating geometrical figures.
- Its coherence. Danielle McNamara and collaborators have shown that text comprehension is mostly determined by its cohesion, i.e. how topics in a text are related to each other [14]. Hence our first driving hypothesis is that the degree of coherence –which is a main factor driving cohesion– should be a very strong indicator of the communication score of a description.
- Whether it was procedural or not. Some pedagogical traditions have placed a strong emphasis in the description of procedures (for instance inclined to use logo like programming in school) [15]. Here we can settle empirically whether descriptions are more effective when weighted towards procedural terms (i.e. first go to the left corner, then draw a straight line…) or containing more mathematical concepts (it is a symmetric triangle, with a square on top).
- Whether it had mathematical content (mainly geometry and number concepts). our current knowledge does not make clear predictions on whether using more mathematical terms or procedural descriptions should be more effective to convey a geometric image. Since, children from a young age have well developed intuitions of number, shape and other geometric features [16, 17], one can argue that using mathematical concepts may capitalize on an intuitive description system.

Our aim is to investigate which of these five aspects of the description (coherence, mathematical-content, procedural, creativity and metaphorical) predicts its capacity to faithfully convey a message.

## Methods and Procedures

### Android Application

This experiment was based in an Android Application. The application was developed by the Mobile Research Team at University of Buenos Aires, and added to the Google Play Store.

**Fig 2:**
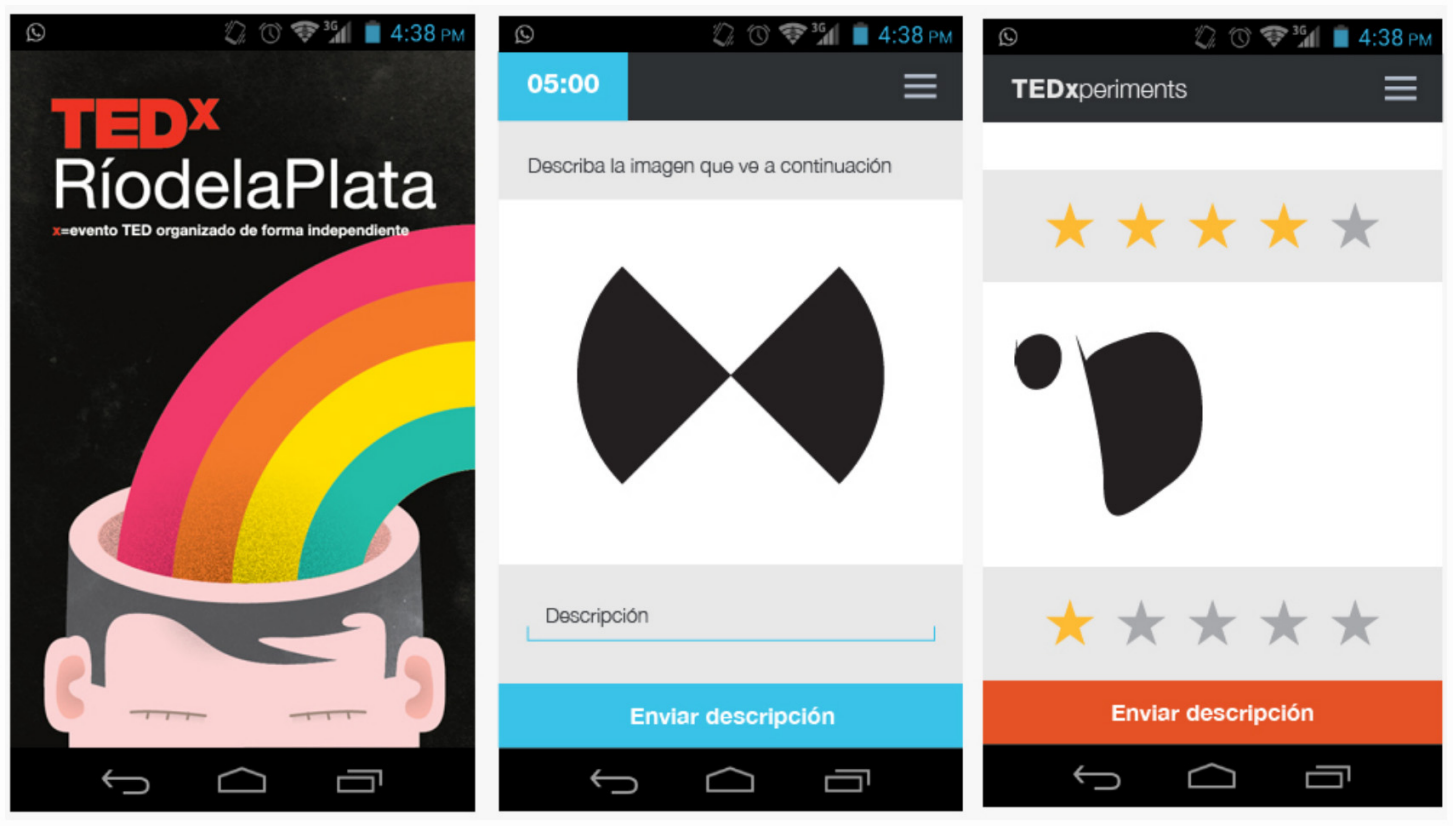
Android App screenshots. The left screenshot is the presentation screen. In the middle, the writing stage is shown. The screenshot in the right shows a rating screen.

The experiment consisted in 3 stages: 1) writing, 2) drawing and 3) rating. Each stage has a written explanation before the subject begins. In Fig 2 we show three screenshots of tha Android App (the presentation screen, the writing-phase screen and the rating-phase screen) as examples.

In the first stage, the writing stage, the subject has the role of *emitter*: receiving a randomly assigned image (there are 4 different categories with 4 different images (see Fig 1a) and writing a text to let another person replicate this picture later. After writing, the second stage is the *description*. Here the subject receives a description produced by another participant. This text was randomly selected with the constraint that was created describing an image of a different category. The subject reads the instructions and then draws in a white canvas. The only two possible actions are draw with black or erase (i.e. draw with white).

Finally in the last stage, the subjects rate the different parts of the process (see Fig 1b). The application selected pictures, descriptions or drawings from other players. Participants never received own material to rank. The three ranking options were:

- Description score: they receive a picture and a description and rate the quality of the text.
- Drawing score: they receive a description and a drawing and rate the quality of the production.
- Communication score: they receive a picture and a drawing and rate the visual similarity of both elements.

Participants also were asked to rate descriptions in different dimensions (see Fig 1c):

- Creativity. A description is creative when it relies on an original idea to describe the image.
- Metaphors. A description is very metaphorical when uses a different semantic context as resources for explanation.
- Coherence. A description is very coherent when it makes sense and is well formed, allowing fluent reading.
- Procedural. A description is procedural if it explicitly presents the steps to get the result. In contrast, a description is not procedural when it does not present steps but a description of how it looks.
- Mathematical. A description is very mathematical or geometrical when it includes terms of these areas.

In contrast to the description and drawing stages where subjects could only do these stages once, the rating stage could be completed as many times as the subject want.

### Participants

We capitalized on the massive event TEDxperiment in 2013 in Buenos Aires, Argentina, with more than 1200 people at TEDxRíodelaPlata (http://www.tedxriodelaplata.org). The TEDxperiments initiative aims to construct knowledge on human communication. During this event, the attendees ran a live experiment during the TEDx Talks in human verbal communication [18].

Before this experiment, registered people to the event were invited to participate in a on-line experiment through an Android App. Subject who decided to participate signed a consent to allow us to use the collected data for research. After signing the consent, participants who accepted the invitation downloaded the Android App2 from the Google Play Store. The procedures of the experiments described here were approved by the ethics committee of CEMIC (Centro de Educación Médica e Investigaciones Clínicas Norberto Quirno).

Approximately 700 people participated in the experiment (for details of age and gender distribution see Table 1).

**Table 1:** Age and sex distribution of the participants.

Age	Male	Female	Total
<20	48	62	110
20–29	202	135	337
30–39	109	62	171
≥40	46	25	71
Total	405	284	689

## Results

From the first stage we collected 689 descriptions. The second stage, the drawing, was completed by 90% of the participants (621 drawings). Participants were engaged with the last stage: we got near 4000 rates for each of the evaluation phases (the communication, the description, the drawing and the speech properties i.e. the five dimensions of the descriptions).

### Correlational structure of the data

Naturally, the different measures are correlated as the communication score combines the precision of the description (*Description score*) and the capacity of the receiver to translate faithfully the description into a drawing (*Drawing score*).

Here we do not seek to dig in detail how the communication score combines from these two individual steps, or how the drawing performance affects the whole communication process. Instead, we merely investigate the natural expectation that the quality of the description is tightly correlated to the quality of the entire communication process, given the fact that drawings are good.

**Fig 3:**
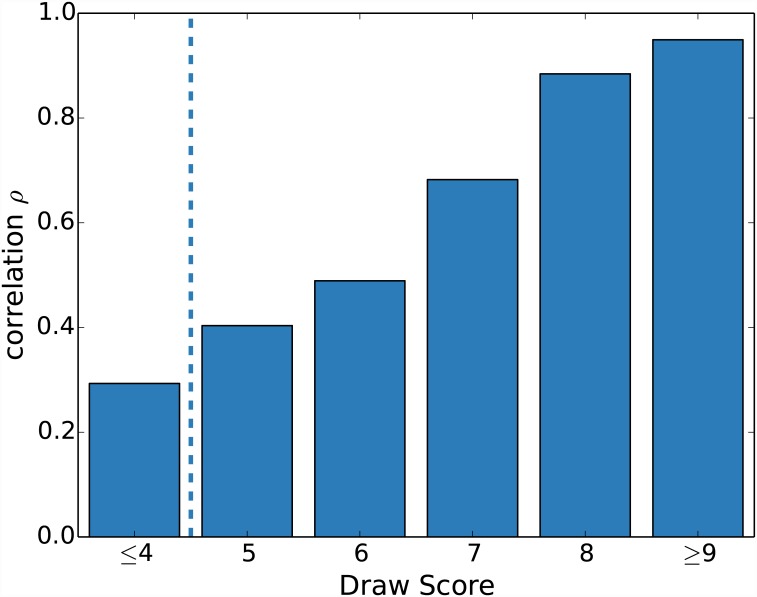
Correlation as function of the draw quality. Correlation *ρ* between *Communication score* and *Description score* for the partition of the dataset for different values of the *Drawing score*.

To this aim, we partition the dataset for different values of the *Drawing score*, i.e. groups of communication and description scores with drawing score between 0 and 1, between 1 and 2, …, between 8 and 9 and higher than 9. For each of these partitions, we measure the correlation between *Communication score* and *Description score*. Results show that the correlation is close to zero for the worse ranked drawings and grows to values close to 1 for the best ranked drawings (Fig 3). As we are interested in characterizing which aspects make a good description, for all subsequent analyses we discard the data with (*Drawing score* ≤ 4).

**Fig 4:**
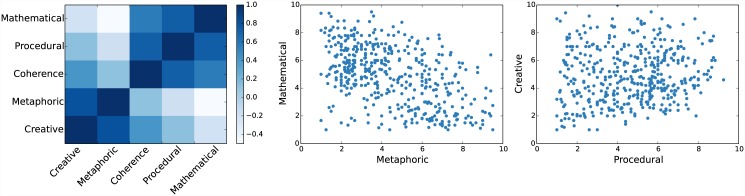
Relation between speech properties. Panel a) shows the *ρ* of Pearsons correlations between every speech properties. Panel b) shows two example of correlation between some speech properties. **(a)** Correlation between all speech properties. **(b)** Examples of correlations.

Next we investigate the correlation matrix of the five speech properties of the description. This result showed that the correlation matrix is organized in two blocks: one corresponding to coherence, procedural and mathematical, and the other to metaphorical and creativity (Fig 4A). However, within these blocks there is sufficient dispersion in the data to assure a stable simultaneous regression to these five attributes (all correlations within blocks are between 0.5 and 0.8, Fig 4B).

### Attributes affecting description and communication scores

Our main aim was to investigate which attributes of a description affect its capacity to faithfully convey an image. For each description and whole communication, we obtained a rating score for each of the five dimensions based on the approximately 3000 ratings by participants (see Table 2).

**Table 2:** Participants ratings on description properties.

Property	#ratings	Average Rating	STD
Creativity	2992	4.949 / 10	2.978
Metaphorical	2982	4.546 / 10	3.161
Coherence	2991	5.407 / 10	2.948
Procedural	2936	4.611 / 10	3.042
Mathematical	3001	4.869 / 10	3.083

**Fig 5:**
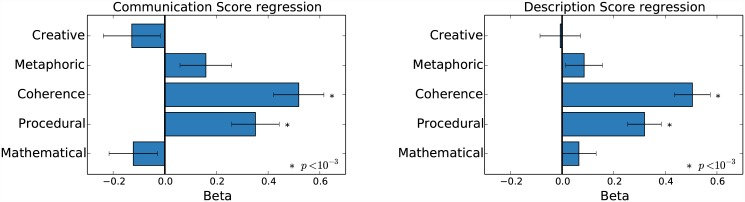
Speech properties linear regression.

We performed two independent linear regressions (one for *Communication score* and one for *Description score*) with these dimensions ratings. Both analyses showed the same result: a highly significant effect of procedure and coherence and no effect of the other three attributes (Fig 5).

## Discussion

Here we capitalized on vast data obtained from an Android App to quantify the effect of different aspects of a description on communication precision. We show that descriptions more effectively communicate an image when they are coherent and when they are procedural. Instead, the creativity, the use of metaphors and the use of mathematical concepts does not affect its fidelity.

The motivation for this experiment was to construct a minimal and controlled setup emulating important variables of pedagogy amenable to quantitative analysis. This follows the strategy used by Shafto and Goodman [19, 20] to inquire whether learners adapt to make optimal inferences from the specific examples provided by teachers. Communication and pedagogy are intrinsically related with only subtle differences [21]. Compared to a typical pedagogical situation this experiment is simplified in three critical parameters: 1) the description is exclusively based on words (there are no gestures, prosody, ostensive indicators, etc. which are known to be fundamental for pedagogy [22, 23]); 2) there is no dialog or conversation, and hence no online feedback or opportunity to correct errors during the pedagogical process. Instead, our setup builds on a one way message resembling or modeling of an expository lecture; 3) the concept to convey is simplified to a simple geometric figure instead of a more elaborated concepts. Thus we propose this as a model of an extremely eroded and simplified version of a minimal pedagogical dialog. The great advantage of this design is that we can replicate many instances of the same pedagogic process and hence inquire which aspects of the message are relevant for the success of the pedagogical experience.

The observation that, when filtered for good drawings, the communication and description scores are highly correlated indicate that participants have a good understanding of what constitutes an effective description. This finding is not trivial since students often rely in heuristics to evaluate the effectiveness of a lecture, leads to misperceptions of the quality of a class. For instance, in the well known “Dr. Fox effect” students rely on teacher expressiveness (often more than on its content) to provide teacher ratings [24]. In our study we reasoned that judges may believe that creative or metaphoric explanations would be more effective than they are in reality. This hypothesis was rejected by showing that participants understand that coherence and procedural content are the most effective speech properties of a description and that, conversely, highly creative or highly metaphoric descriptions are not very likely to be effective.

Many possible hypothesis raise to explain why creativity and metaphorical descriptions are less effective than coherent and procedural ones. On one hand, selected geometrical figures lack of emotional content. Creativity and metaphorical descriptions may be more effective where complex images with contented feelings or emotionally relevant scenes are to be transmitted. Another possible hypothesis may be that communication protocol of experiment affects the effectiveness of different speech properties. Creative and metaphorical descriptions may need of two-way communication channel allowing interaction—dialogs—between participants, instead of the one-way protocol adopted in this experiment. These alternative hypothesis could be tested by variants of the presented experiment, where different (and tagged) images should be selected or adopting a communication protocol which allows asking for additional details of the description.

It is important to mention that in our game communication was considered effective when the resulting drawing replicated accurately the original image. In other pedagogical contexts where the goal may not be to optimize the precision of the communicated message, the results are expected to change.
